# Method for computer-aided modeling of water adsorption isotherms in Achira biscuits (*Canna edulis* K.)^☆^

**DOI:** 10.1016/j.mex.2025.103614

**Published:** 2025-09-08

**Authors:** Gentil A. Collazos-Escobar, Andrés F. Bahamón-Monje, Nelson Gutiérrez-Guzmán

**Affiliations:** a Grupo de Análisis y Simulación de Procesos Agroalimentarios (ASPA), Instituto Universitario de Ingeniería de Alimentos–FoodUPV, Universitat Politècnica de València, Camí de Vera s/n, Edificio 3F, València 46022, España; b Centro Surcolombiano de Investigación en Café (CESURCAFÉ), Departamento de Ingeniería Agrícola, Universidad Surcolombiana, Neiva-Huila 410001, Colombia.; c Departamento de Ingeniería Agroindustrial, Facultad de Ingeniería, Universidad Surcolombiana, Neiva, Huila, Colombia

**Keywords:** Model-based investigation, Mathematical modeling, Optimization, Surface response

## Abstract

Mathematical modeling of water adsorption isotherms (WAI) plays a fundamental role in food engineering, enabling the quantitative description and prediction of moisture sorption behavior in food systems. Despite its relevance, there is a notable gap in the literature concerning computer-aided methods for fitting WAI data. This study aims to fill that gap by introducing an advanced computational approach for modeling WAI using three empirical equations: Peleg, Polynomial, and Double Log Polynomial (DLP). A comprehensive experimental dataset was used, consisting of WAI measurements from Achira biscuits over a wide range of water activities (a_w_: 0.1 to 0.9) and storage temperatures (25, 35, and 45 °C). The predictive performance of each model was assessed using statisticalgoodness-of-fit metrics, including root mean square error (RMSE), coefficient of determination (R²), and adjusted R², supported by graphical analysis. All models achieved excellent fitting results, with RMSE values below 5 % and R² and R²_adj_ consistently above 99 %. These findings validate the effectiveness of the computer-aided modeling framework for accurately describing WAI behavior in low-moisture foods. Furthermore, the study demonstrates the potential of this approach as a practical and reliable tool for optimizing storage conditions, enhancing moisture control, and supporting decision-making in food quality and shelf-life management.

This study:

• Enables the statistical modeling of WAI.
• Proposes an advanced framework for the computer-aided procedure of WAI.

## Graphical abstract

**Figure:**
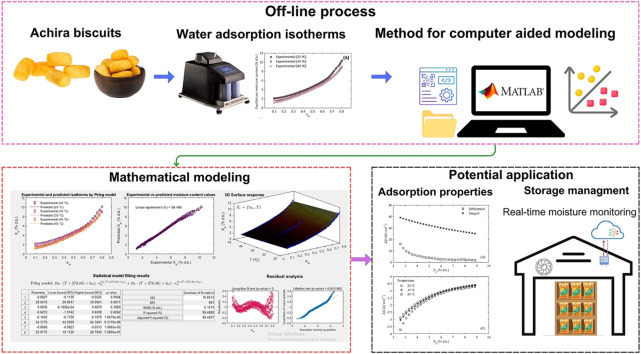

**Specifications table**

**Table:** 

**Subject area**	Food Science
**More specific subject area**	Mathematical modeling
**Name of your method**	Empirical modeling of water sorption isotherms
**Name and reference of original method**	Model-based investigation of water adsorption in Achira (*Canna edulis* K.) biscuits [1] Water dynamics adsorption properties of dried and roasted cocoa beans (*Theobroma cacao* L.) [2] MODELING DYNAMIC ADSORPTION ISOTHERMS AND THERMODYNAMIC PROPERTIES OF SPECIALTY GROUND ROASTED-COFFEE (*Coffee Arabica* L.) [3] Moisture sorption isotherms and thermodynamic properties of tiger nuts: An oil-rich tuber [4] Effect of temperature on moisture sorption isotherm characteristics of Thai jasmine paddy (Khao Dawk Mali 105) [5]
**Resource availability**	The data and MATLAB code that supports the findings of this study are available within the: Repository name: Mendeley Data Data identification number: 10.17632/t25r4r7nw8.2 Direct URL to data: https://data.mendeley.com/datasets/t25r4r7nw8/2

## Background

Water sorption isotherms (WSI) are recognized as essential tools within the food industry [6], as they offer indispensable insights into the moisture behavior of food products and their interaction with environmental humidity [7]. The WSI describes the relationship between water activity (a_w_) and equilibrium moisture content (X_e_) at a constant temperature, rendering them reliable for numerous applications in food engineering [2]. A primary application of WSI is the assessment of a_w_, a significant parameter influencing microbial growth, enzymatic activity, and chemical reactions in food. By comprehending the X_e_ associated with particular a_w_ levels, food manufacturers can predict and regulate spoilage and quality degradation, ensuring food safety and prolonging food-shelf life. WSI play a key role in determining storage stability [8], facilitating predictions of moisture changes during storage and guiding the design of packaging solutions to prevent undesirable moisture gain or loss. Furthermore, WSI enable the estimation of shelf life by identifying critical moisture levels that trigger spoilage or cause physical changes in products [9].

Mathematical modeling is a powerful tool for investigating and comprehending intricate manufacturing processes [1]. Precise mathematical modeling of experimental WSI facilitates the estimation of effective surface area, spreading pressure, and pore radius, as well as thermodynamic adsorption properties such as differential and integral enthalpy/entropy and Gibbs free energy [10]. These properties are critical for elucidating water-binding mechanisms and can be utilized as reliable criteria for predicting the storage stability of dehydrated food products [11].

Recent studies have highlighted the significance of accurate WSI modeling in enhancing food quality and safety. For example, Nayab et al. [12] demonstrated how WSI analysis can be used to improve packaging design and shelf-life prediction in dehydrated fruit products, while Zhang et al. [4] emphasized the role of sorption modeling in the development of moisture control systems for foods. Such research underlines the growing reliance on WSI for data-driven decision-making in modern food engineering. Nevertheless, although WSI modeling proves highly useful, there are still limited computational frameworks that are readily accessible to non-expert users in the field of food science. It remains a complex task requiring advanced mathematical knowledge and expertise in computational programming. This complexity poses significant barriers for food scientists and manufacturers, limiting their ability to leverage robust computational tools for real-time decision-making in the food industry.

The novelty of this work lies in the development of a computational modeling approach specifically tailored for non-specialist users, demonstrated through its application to a low-moisture product such as Achira biscuits, used here as a representative case study. In contrast to previous studies that largely emphasize model fitting or empirical validation, this research introduces a reproducible and user-friendly pipeline that effectively bridges the gap between theoretical modeling and practical application. By integrating advanced statistical techniques into an accessible computational framework, our methodology empowers food professionals to independently perform WSI modeling and shelf-life estimation without requiring extensive programming expertise. This approach enables users to model WSI effectively while leveraging state-of-the-art statistical techniques. By addressing the aforementioned challenges, this work contributes to the practical application and widespread availability of these advanced decision-making models in the food industry. Beyond its application to Achira biscuits, this methodology establishes a scalable framework that can be extended to other food products. The versatility of this methodology renders it a valuable instrument for researchers and industry stakeholders seeking to optimize food processes and enhance product quality.

Therefore, the main aim of this work was to develop an advanced computational methodology, supported by a detailed user guide, for modeling WSI in low-moisture food products, providing a scalable framework adaptable to water sorption analysis across a wide range of food products.

## Method details

**Fig. 1:**
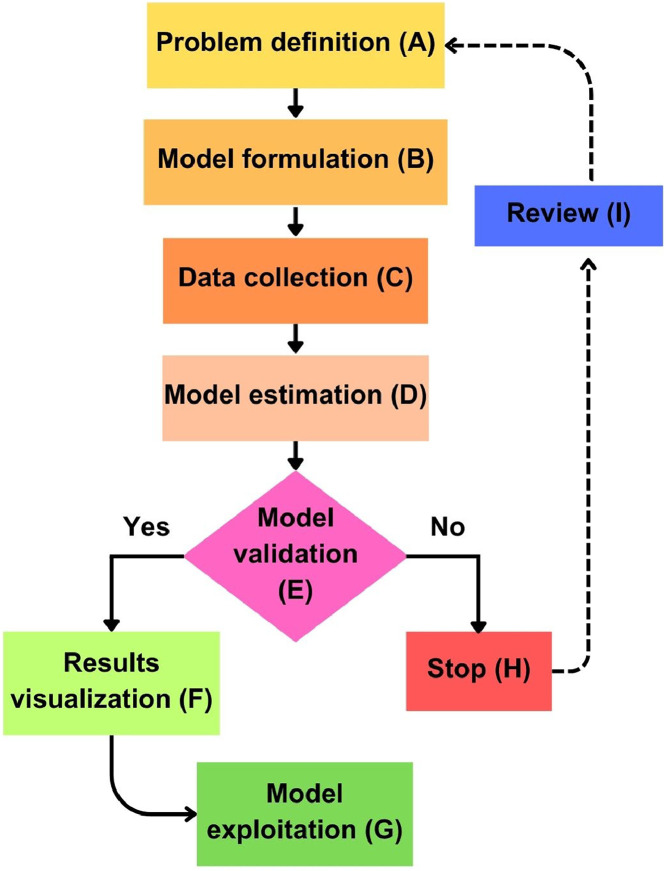
Flowchart illustrating the classical procedure used for calibrating a mathematical model. The steps involved are problem definition (A), model formulation (B), data collection (C), model estimation (D), model validation (E), results visualization (F), and model exploitation (G). If the model is not correctly validated, steps H, I, and A are followed to refine the process.

The methodology established for the computer-aided modeling of WAI in Achira biscuits (*Canna edulis K.*) followed a framework comprising seven principal stages: problem definition, model formulation, data collection, model estimation, model validation, results visualization, and model exploitation. These stages are summarized in the MATLAB® R2023a (The MathWorks Inc., Natick, MA, USA) code **“MathematicalModelingIsotherms.m”**. Every step is outlined in the flowchart provided in Fig. 1 and detailed in the subsequent sections.

### Problem definition

The first step involved in any data analytic procedure is defining the scope and objectives of the modeling process (Fig. 1A). For this study, the main aim was to develop a computational tool to assist the calibration of different mathematical models (Peleg, Polynomial, and DLP) for accurately describing the WAI of Achira biscuits. The focus was on describing the relationship between a_w_ and X_e_ at different temperature conditions. This analysis provides valuable insights into the water sorption properties of this product, facilitating better monitoring and control of storage stability and shelf life.

### Model formulation

In order to address the computer modeling of WAI (Fig. 1B), a mathematical expressions able to describe the influence of a_w_ on X_e_ were considered. Thereby, sorption equations such as Peleg (Eq. 1), Polynomial (Eq. 2), and DLP ((3), (4)) were used based on their tested reliability in describing WSI in food products [[1], [2], [3],^7^,^13^,^14^]. Each model involves specific parameters (b_i_, i=0-3) that define the functional relationship between a_w_ and X_e_. These models were chosen for their simplicity, minimal number of parameters, and ability to effectively describe non-linear relationships between variables [15] and they have demonstrated their feasibility in the mathematical description of WSI in foods [[7], [16], [17], [18], [19], [20]]. Additionally, the method for computer-aided modeling is flexible, allowing users to include as many models as desired to characterize WSI. While we have presented the Peleg, Polynomial, and DLP models as examples, new models can be easily incorporated depending on the user’s specific aims. In particular, additional models for the mathematical description of WAI can be integrated into the “MathematicalModelingIsotherms.m” MATLAB code.

(1) $$X_{e}=b_{0}{a_{w}}^{b_{1}}+b_{2}{a_{w}}^{b_{3}}$$

(2) $$X_{e}=b_{0}+b_{1}a_{w}+b_{2}{a_{w}}^{2}+b_{3}{a_{w}}^{3}$$

(3) $$X_{e}=b_{0}+b_{1}x+b_{2}x^{2}+b_{3}x^{3}$$

(4) $$x=\text{ln}(-\ln a_{w})$$

In order to assess the temperature (T; K) influence in the mathematical modeling using Peleg, Polynomial and DLP models, the temperature was linearly linked to each model’s parameter according to reported by [3]. Thus, the generalized Peleg (Eq. 5), Polynomial (Eq. 6) and DLP (Eq. 7) were used as formulated models as follow.

(5) $$X_{e}=\left(b_{0}T+b_{01}\right){a_{w}}^{\left(b_{1}T+b_{11}\right)}+\left(b_{2}T+b_{21}\right){a_{w}}^{\left(b_{3}T+b_{31}\right)}$$

(6) $$X_{e}=\left(b_{0}T+b_{01}\right)+\left(b_{1}T+b_{11}\right)a_{w}+\left(b_{2}T+b_{21}\right){a_{w}}^{2}+\left(b_{3}T+b_{31}\right){a_{w}}^{3}$$

(7) $$X_{e}=\left(b_{0}T+b_{01}\right)+\left(b_{1}T+b_{11}\right)\text{ln}\left(-\ln a_{w}\right)+\left(b_{2}T+b_{21}\right)\text{ln}{\left(-\ln a_{w}\right)}^{2}+\left(b_{3}T+b_{31}\right)\text{ln}{\left(-\ln a_{w}\right)}^{3}$$

The mathematical models were included in the MATLAB code **MathematicalModelingIsotherms.m** by using the “fittype” MATLAB function, and were subsequently used as explained in Model estimation section.

### Data collection

Data collection (Fig. 1C) is one of the most important steps in a data analysis process since a successful model-based investigation highly depends on the quality and accuracy of the experimental data used to calibrate the model [21]. Therefore, in light of calibrating the sorption models as well as possible, a comprehensive dataset with an elevated number of experimental data points was considered. In this sense, the dataset **“Experimental water adsorption isotherms and Attenuated Total Reflectance-Fourier Transform Infrared (ATR-FTIR) analysis in Achira (*Canna edulis* K.) biscuits”** was used as the basis for the development and validation of the method for computer-aided modeling of WAI. The experimental data were obtained from commercial Achira biscuit samples purchased in a local store in Neiva (Huila region, Colombia). The biscuits used for laboratory analysis were produced using Achira starch (270 g, 30.86 % w/w), fresh cheese (500 g, 57.15 % w/w), butter (50 g, 5.71 % w/w), eggs (50 g, 5.71 % w/w), and salt (5 g, 0.57 %). The ingredients were first mechanically mixed, and the dough was manually shaped into biscuits. Baking was carried out at 200 °C for 20 minutes. After baking, the biscuits were tempered at ambient conditions (25 °C, 40 % relative humidity) for 2 hours and subsequently packaged in 250 g polypropylene bags. Then, the WAI was determined by using the automatic dynamic dewpoint isotherm (DDI) method employing a vapor sorption analyzer (VSA Aqualab Decagon Devices, Inc. Pullman, WA), considering a wide range of a_w_ between 0.1 to 0.8, at different temperatures of 25, 35, and 45°C. The a_w_ point in every replicate (n=3) of WAI was acquired considering a resolution of 0.01 a_w_ [1]. Before the measurements, the VSA equipment was calibrated using three different saturated salt solutions provided by instrument manufacturers (LiCl: concentration of 13.41 mol/kg; a_w_= 0.250 ± 0.003 and concentration of 8.57 mol/kg; a_w_=0.500 ± 0.003, and NaCl: concentration of 6.0 mol/kg; a_w_=0.760 ± 0.003). As a result, a high number of experimental data points per each replicate (>75 points) was obtained [20,22]. The elevated number of sorption data points increased the precision in the description of the real process, which is essential for subsequent robust computer modeling [23]. The feasibility of the DDI method for the analysis of WSI has been successfully demonstrated by [[2], [20], [22], [24]].

**Fig. 2:**
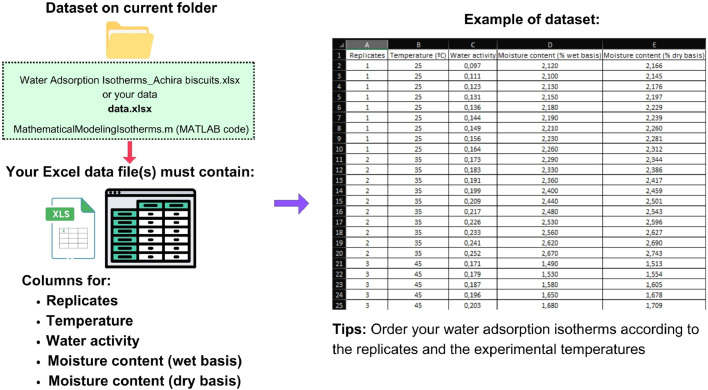
Data accessibility procedure for computer modeling of water adsorption isotherms.

An example of data accessibility of WAI of Achira biscuits and tips on organizing any dataset for its use in the MATLAB code are described in Fig. 2

### Model estimation

The statistical procedure for model fitting of WAI (Fig. 1D) was carried out by formulating and solving an optimization problem. For this goal, the mathematical models (Model formulation section; Fig. 1B) served as a virtual representation of the relationship between X_e_, a_w__,_ and T, and their model parameters were considered as the decision variables that quantify the influence of a_w_ and T on X_e_. Therefore, a non-linear least squares regression analysis was conducted using MATLAB, wherein the decision variables (b_i_ parameters) and sorption models ((1), (2), (3), (4)) were used to minimize the Mean Square Error (MSE, Eq. 8) as the objective function in this optimization process.

(8) $$\text{MSE}=\frac{{\left(X_{e}-\;X_{\text{epred}}\right)}^{2}}{N}$$

Where X_e_ and X_epred_ are the experimental and predicted equilibrium moisture content values ( % d.b.) and N in the number of experimental data points.

The model fitting procedure and the configuration of the optimizer were conducted using the “fit” and “fitoptions” MATLAB functions, respectively. The optimization process was achieved using the Levenberg-Marquardt method, which was employed to find the optimal values of decision variables [25]. Furthermore, by using the “fitoptions,” the tolerance criteria (1 × 10^–6^), the algorithm is constrained to a maximum of 600 evaluations and 400 iterations were defined to ensure precision in convergence and control of computational complexity (in any case, the user could try the model fitting by changing these values). The optimization algorithm iteratively adjusts the b_i_ parameters until convergence is achieved, ensuring that the model fits the data accurately. The statistical estimation of significance in model parameters was performed using the t-student statistical test, and their confidence intervals (95 %) were calculated using the “confint” function of the MATLAB software.

**Fig. 3:**
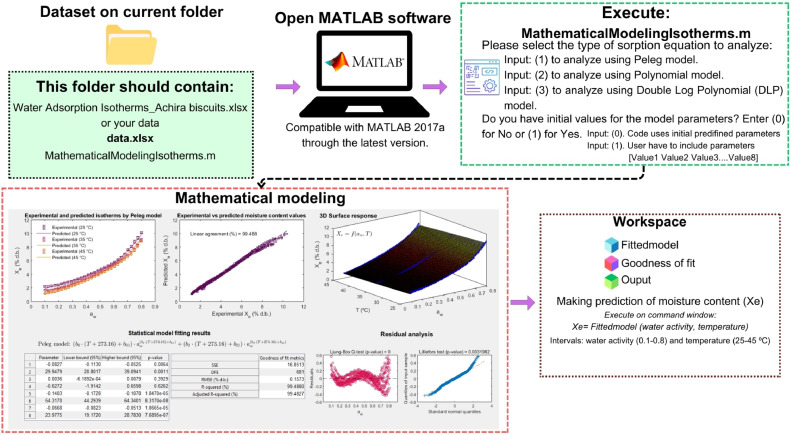
Step-by-step assistance procedure for modeling water adsorption isotherms in achira biscuits, guiding users through the selection of the sorption equation, input of initial model parameters, generation of fitting results, and prediction of equilibrium moisture content.

The initial values of decision variables were predefined in the code as a row vector [1]. These values were reported as suitable for a well-fitting of WAI in Achira biscuits using Peleg, Polynomial, and DLP models, and they also could be useful for modeling WSI in other food products. However, if the parameters for Achira biscuits do not successfully fit the modeling of WSI in other products, or if the user intends to define custom values, the MATLAB code accommodates this flexibility. In such cases, the b_i_ parameters must be specified following the example provided in Fig. 3.

Upon executing the file **MathematicalModelingIsotherms.m** in the MATLAB editor, the program loads the experimental dataset and assigns the names of variables (replicates, temperature, water activity, and moisture content) to the corresponding columns in the Excel file. Then, the code prompts the user to select the type of sorption model for analysis (Fig. 3). A user input of 1 runs the Peleg model, an input of 2 uses the Polynomial model, and an input of 3 uses the DLP equation. Afterwards, the program solicits users to declare if the initial value of model parameters are known (input 1). These values should be introduced in the format: [value1 value2 value3 value4 value5 value6 value7 value8], separated by spaces and without commas. Conversely, the program uses those predefined values for Achira biscuits. The MATLAB program runs the code, resulting in an interface with the fitted model, statistical and visualization results, and the calibrated model to be further used in the prediction of the equilibrium moisture content of Achira biscuits or the food product assessed (Fig. 3).

### Model validation

The validation process (Fig. 1E) assessed the predictive ability of calibrated sorption models in the description of WAI in Achira biscuits. During this process, the feasibility of optimized model parameters (b_i_ as decision variables) are evaluated by measuring the performance of the predicted X_e_ using these parameters. The predictive performance of sorption models was analyzed by computing the goodness of fit metrics such as RMSE (Eq. 9), R^2^ (Eq. 10) and R^2^_adj_ (Eq. 11).

(9) $$\text{RMSE}=\sqrt{\text{MSE}}$$

(10) $$R^{2}\left(\%\right)=100-\frac{\sum_{i=1}^{N}{\left(X_{e}-\;X_{\text{epred}}\right)}^{2}}{\sum_{i=1}^{N}{\left(\bar{X_{e}}-\;X_{\text{epred}}\right)}^{2}}$$

(11) $$R_{\text{adj}}^{2}\left(\%\right)=100-\left(\frac{N-1}{N-M}\right)\left(100-R^{2}\right)$$

Where $\bar{X_{e}}$ is the mean of X_e_ and M is the number of model’s parameters. These metrics were adequate to define if a calibrated model can be considered as reasonable for practical applications in real scenarios (model with lower figures of RMSE and R^2^_adj_ >98 %) [26]. If the models parameters failed to achieve acceptable accuracy in the prediction of X_e_ (e.g., high RMSE or low R^2^ and R^2^_adj_), the tool provides feedback (Fig. 1H, 1I) for revisiting earlier steps (Fig. 1A) such as parameter optimization. Conversely, if the model parameters were adequately estimated, the results of sorption model calibration could be considered as validated (Fig. 1E). Furthermore, the residuals of models were computed as the difference between X_e_ and X_epred_. Then, they were summited to an independence test (using the Ljung-Box Q-test; “lbqtest” MATLAB function) and normality test (using the Lilliefors test; “lillietest” MATLAB function).

### Results visualization

**Fig. 4:**
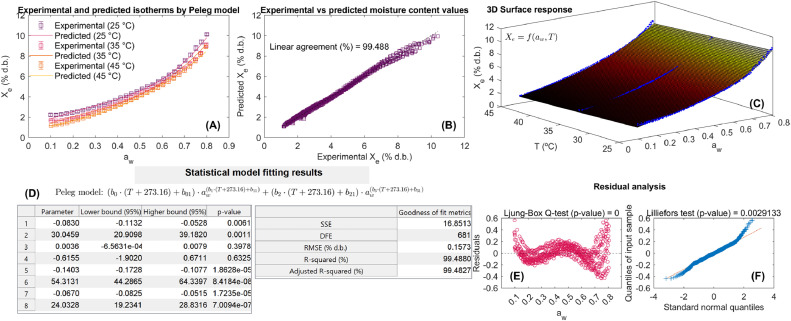
Statistical results of method developed for computer modeling-aided modeling of water adsorption isotherms (WAI). Experimental and predicted WAI at different temperatures (A), agreement between experimental and predicted equilibrium moisture content values (B), 3-Dimensional surface representation of experimental and predicted WAI at different temperatures (C), model fitting results including model parameters, confidence intervals, statistical significance of model parameters and goodness of fit metrics of fitted model (D), and analysis of model residuals: independence (E) and normality (F) tests.

The visualization of results (Fig. 1F) is a critical step in any data analysis process, as it enables precise interpretation of model performance and highlights areas where adjustments could improve the predictive accuracy of models. To support the clear and efficient representation of the statistical results of the method for computer-aided mathematical modeling of WAI, an interface was developed (Fig. 4). This interface enables users to identify the main results of the models, facilitates a clear interpretation of modeling performance, and supports refinements to improve the accuracy and reliability of calibrated sorption models.

The statistical results of the developed method for computer-aided modeling of WAI are comprehensively presented in several dimensions. These include: the comparison of experimental and predicted WAI across different temperatures and a_w_ levels, illustrating the model’s predictive capabilities (Fig. 4A); the degree of agreement between experimental and predicted equilibrium moisture content values, providing a quantitative validation of the model (Fig. 4B); a 3-dimensional surface representation of experimental and predicted WAI at different temperatures, enabling a visual assessment of model alignment with experimental data (Fig. 4C); detailed model fitting results, encompassing model parameters, confidence intervals, statistical significance of parameters, and goodness-of-fit metrics, which assess the robustness and reliability of the fitted model (Fig. 4D); and analysis of model residuals through independence (Fig. 4E) and normality (Fig. 4F) tests, ensuring that the residuals meet the underlying assumptions of the statistical model. In the case of Fig. 4A, as the experimental dataset on WAI in Achira biscuits provides replicates of WAI at all temperatures, the MATLAB code calculates the mean and standard deviation of replicates for each temperature to represent the variability of the WAI. Additionally, the 3D surface response results of the calibrated mathematical models in Fig. 4C were plotted using the “meshgrid,” “surf,” and “scatter3” MATLAB functions.

### Model exploitation

Following the calibration and validation of the mathematical sorption models, the final step in the computer-aided procedure is model exploitation (Fig. 1G). In this sense, the statistical outcomes derived from the interface (Fig. 4) establish the foundation for model-based interpretation of water adsorption phenomena. The analysis of b_i_ parameters may elucidate the influence of a_w_ and T on X_e_. In this step, the calibrated models could also be used to make predictions of X_e_ as a function of a_w_ and T. Given that all the experimental conditions were modeled via the sorption models, it is possible to predict X_e_ within the boundaries of a_w_ and T (0.1-0.8 a_w_ and 25-45°C). To achieve this objective, the MATLAB code stores an object in the MATLAB software workspace. This object is saved with the name of “**Fittedmodel”** and can be directly used in the command window of the MATLAB program to predict moisture content in food product as a function of a_w_ and T (Fig. 3). In order to predict X_e_, it is necessary to use the following syntax: **Fittedmodel(a_w_ value, T value)**. It is imperative to note that the predictions are contingent upon the boundaries of a_w_ and T that are incorporated within the mathematical framework. Beyond these limits, the model predictions are subject to inaccuracies. To illustrate this, consider the prediction of X_e_ at a_w_ = 0.55 and T = 28 °C. The syntax for this prediction is **Fittedmodel(0.55, 28)**. Upon entering this into the command window, the predicted X_e_ result is displayed. The tool enables users to predict moisture content under specific conditions of a_w_ and T, facilitating real-time decision-making in food processing and storage. The scalable framework also enables the extension of this methodology to other food products by simply inputting new datasets and recalibrating the sorption models.

### Method validation

**Fig. 5:**
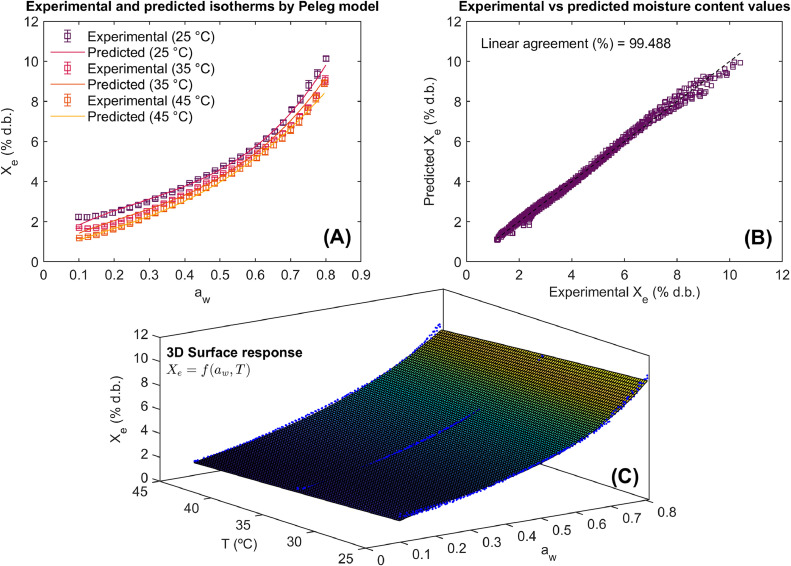
Experimental and predicted water adsorption isotherms (WAI) at different temperatures (A), correlation between experimental and predicted values using the Peleg model (B), and 3D representation of experimental WAI of Achira biscuits with the surface response modeled by the Peleg equation (C).

**Fig. 6:**
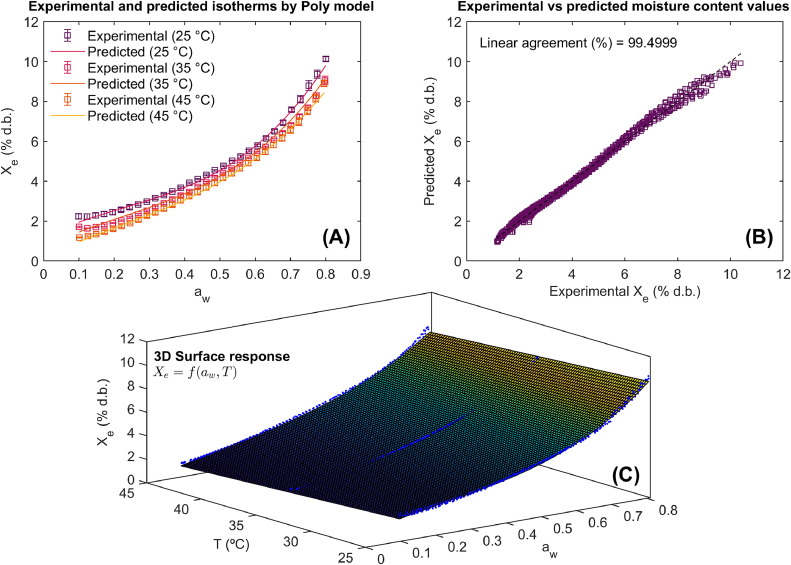
Experimental and predicted water adsorption isotherms (WAI) at different temperatures (A), correlation between experimental and predicted values using the Polynomial model (B), and 3D representation of experimental WAI of Achira biscuits with the surface response modeled by the Polynomial equation (C).

**Fig. 7:**
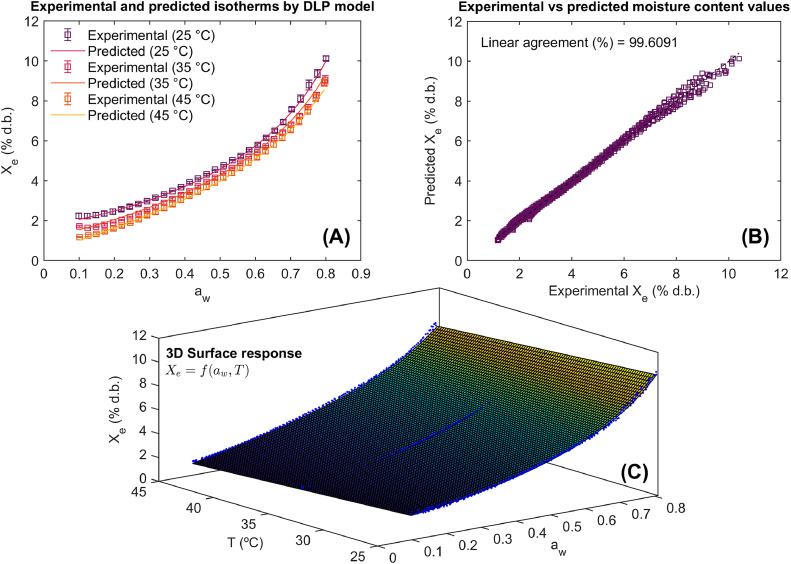
Experimental and predicted water adsorption isotherms (WAI) at different temperatures (A), correlation between experimental and predicted values using the DLP (Double Log Polynomial) model (B), and 3D representation of experimental WAI of Achira biscuits with the surface response modeled by the DLP equation (C).

The statistical results of the use of method for computer-aided modeling of WAI in Achira biscuits are illustrated in Table 1 and Fig. 5, Fig. 6, Fig. 7.

**Table 1:** Results of statistical model fitting for adsorption isotherms in roasted specialty coffee.

Model	Parameters	Confidence intervals (95 %)	Goodness of fit
Peleg	b_0_= –0.083 b_01_= 30.046 b_1_= 3.611×10^–3^ b_11_= –0.615 b_2_= –0.143 b_21_= 54.315 b_3_= –0.067 b_31_= 24.034	[–0.113, –0.053] [20.911, 39.183] [–6.567×10^–4^, 7.912×10^–3^] [–1.902, 0.671] [–0.173, –0.108] [44.288, 64.341] [–0.083, –0.052] [19.235, 28.833]	RMSE= 0.157 % d.b. R^2^= 99.488 % R^2^_adj_= 99.483 %
Polynomial	b_0_= –0.051 b_01_= 16.516 b_1_= 6.411×10^–3^ b_11_= 10.753 b_2_= 0.294 b_21_= –103.131 b_3_= –0.381 b_31_= 135.904	[–0.066, –0.037] [12.035, 20.998] [–0.127, 0.114] [–26.548, 48.053] [1.112×10^–3^, 0.587] [–193.431, –12.831] [–0.594, –0.167] [70.083, 201.725]	RMSE= 0.156 % d.b. R^2^= 99.495 % R^2^_adj_= 99.494 %
DLP	b_0_= –0.033 b_01_= 13.309 b_1_= –0.014 b_11_= 1.719 b_2_= –0.0157 b_21_= 5.803 b_3_= 5.712×10^–3^ b_31_= –1.721	[–0.035, –0.031] [12.691, 13.929] [–0.0183, –0.011] [0.566, 2.871] [–0.022, –9.511×10^–3^] [3.903, 7.703] [4.161×10^–4^, 0.011] [–3.354, –0.089]	RMSE= 0.137 % d.b. R^2^= 99.609 % R^2^_adj_= 99.605 %

b_i_ (model parameters), DLP (Double Log Polynomial), RMSE (root mean square error), R^2^ (coefficient of determination), R^2^_adj_ (adjusted coefficient of determination).

The statistical results are shown for each sorption equation as well as their goodness of fit. In the same way, the ability of these models to describe the WAI of the Achira product is represented via the agreement between experimental and predicted values and model fitting in 2D and 3D surface response. The RMSE, R^2^^,^ and R^2^_adj_ were the statistical goodness of fit metrics to assess the predictive power of the Peleg, Polynomial, and DLP calibrated models.

Modeling results and the significance of model parameters (Table 1) demonstrated the ability of the Peleg, Polynomial, and DLP models to describe WAI due to their goodness of fit metrics reaching R^2^ and R^2^_adj_ > 99 % and RMSE <5 %. According to these goodness of fit metrics, it was possible to order the models from highest performance to lowest performance as DPL > Polynomial > Peleg. In any case, all models exhibited greater performance in predicting the WAI in Achira biscuits. This fact can be observed in Fig. 5, Fig. 6, Fig. 7. The Peleg model (Fig. 5A and 5C), Polynomial (Fig. 6A and 6C) and DLP (Fig. 7A and 7C) successfully represent both the type III upward J-concave sorption shape of WAI in Achira biscuits and the temperature influence on the WAI. A close agreement between the experimental and predicted X_e_ was found (Fig. 5B, 6B and 7B), indicating that the prediction of the experimental response was quite similar.

Although the statistical performance of the models was high, a deeper comparative analysis reveals subtle differences in their predictive behavior. The DLP model consistently outperformed the Polynomial and Peleg models, not only in terms of R^2^ and RMSE values but also in capturing the non-linear response of WAI in Achira biscuits across varying temperatures [16]. Notably, the Peleg model tended to slightly underestimate WAI at higher temperatures (Fig. 5), which may be attributed to its simpler mathematical structure and limited flexibility. Conversely, the DLP model exhibited greater robustness in fitting the experimental data, particularly in regions where the sorption curve showed pronounced curvature (Fig. 7). This comparative assessment highlights the advantage of employing more flexible models, such as DLP, when modeling complex response surfaces like the WAI behavior in Achira biscuits.

Beyond the statistical significance of sorption model fitting, the modeling results have practical implications for the optimization of storage conditions and shelf life of Achira biscuits. Accurate prediction of WAI under varying temperature and moisture conditions enables manufacturers to forecast textural changes associated with moisture migration during storage. In particular, the strong performance of the DLP model provides a reliable tool for simulating sorption behavior, facilitating the identification of critical temperature-humidity ranges where WAI increases sharply, potentially compromising product crispness and sensory quality. By integrating this predictive capacity into storage protocols, producers can establish optimal packaging systems and environmental conditions that minimize moisture uptake, thereby extending shelf life and preserving product integrity over time. These findings offer a valuable foundation for the development of moisture-controlled storage strategies specifically tailored to Achira-based products.

Finally, the results obtained using the method for computer-aided modeling of WAI in Achira biscuits demonstrated its reliability in calibrating robust sorption models to predict X_e_ under various storage conditions. Moreover, the quality of the statistical results confirmed the suitability of this method in addressing the lack of standardized approaches in the literature for computer modeling of WAI in Achira. This method also shows potential for application in modeling other food products.

### Limitations

This study represents the first application of a statistical method for computer-aided mathematical modeling WAI in Achira biscuits. It marks an initial step toward developing a robust computer-aided methodology for modeling isotherms in a wide range of agricultural food products. Future research should explore the development of statistical metaheuristic methods, such as genetic algorithms, to optimize the estimation of initial model parameters for their further use in this method. While this study employed parameters suitable for Achira biscuits, it is possible that these parameters may not be universally applicable to other agricultural food products or types of water sorption (adsorption/desorption and different isotherm shapes according to Brunauer-Emmett-Teller-BET classification). Therefore, an optimized method capable of accurately estimating initial model parameters across diverse food types is highly necessary.

Further studies should prioritize the mathematical modeling of multivariate processes, given the alignment of modern food industry practices with the principles of Industry 4.0. In this context, the increasing availability of detailed food processing data necessitates advanced modeling approaches. Models that incorporate additional variables beyond a_w_ and temperature, such as food formulation, processing methods, and other key factors, are crucial for addressing the complexity of food systems. The development of advanced statistical methodologies to generalize current sorption models and integrate these additional variables would significantly enhance their robustness and predictive accuracy. These advancements will enable the creation of more reliable mathematical tools, facilitating better decision-making and process optimization in industrial applications.

## Ethics statements

The method gathered in this work did not involve human subjects, animal experiments, or data obtained from social media platforms.

## CRediT authorship contribution statement

**Gentil A. Collazos-Escobar:** Conceptualization, Methodology, Software, Data curation, Visualization, Writing – original draft. **Andrés F. Bahamón-Monje:** Supervision, Writing – review & editing, Funding acquisition. **Nelson Gutiérrez-Guzmán:** Methodology, Data curation, Supervision, Writing – original draft, Writing – review & editing, Funding acquisition.

## Declaration of competing interest

The authors declare that they have no known competing financial interests or personal relationships that could have appeared to influence the work reported in this paper.

## Data Availability

The data and code used in the preparation of this document have been shared and are accessible at: 10.17632/t25r4r7nw8.2
